# SIRT1 Mediates Neuropathic Pain Induced by Sciatic Nerve Chronic Constrictive Injury in the VTA-NAc Pathway

**DOI:** 10.1155/2020/4245968

**Published:** 2020-08-18

**Authors:** Yangyang Li, Lei Wang, Guotao Zhang, Xueli Qiao, Mingxing Zhang

**Affiliations:** ^1^Department of Anesthesiology, The Affiliated Huai'an No. 1 People's Hospital of Nanjing Medical University, Huai'an 223001, China; ^2^Department of Anesthesiology, The Affiliated Huai'an Hospital of Xuzhou Medical University and the Second People's Hospital of Huai'an, Huai'an 223001, China; ^3^Department of Pharmacy, The Affiliated Huai'an Hospital of Xuzhou Medical University and the Second People's Hospital of Huai'an, Huai'an 223001, China

## Abstract

**Background:**

Mounting evidence has shown that sirtuin 1 (SIRT1), a class III histone deacetylase, alleviated several types of neuropathic pain in the spinal cord and dorsal root ganglion and regulated some aberrant behaviors in the ventral tegmental area (VTA) and the nucleus accumbens (NAc).

**Methods:**

In this context, the effect of SIRT1 on neuropathic pain in the VTA-NAc pathway was investigated in the model of chronic constrictive injury (CCI).

**Results:**

SIRT1 was localized in the VTA neurons in naive mice. The expression of SIRT1 was decreased in the contralateral VTA of CCI mice. After microinjection of SRT1720 (an activator of SIRT1) in the contralateral VTA of CCI mice, the established thermal hyperalgesia was attenuated. However, it was further exacerbated by EX-527 (an inhibitor of SIRT1). The elevated level of acetyl-histone 3 was reduced by SRT1720 but further elevated by EX-527 in the contralateral VTA of CCI mice. The increased expression of Fos in both VTA and NAc was downregulated by SRT1720 but further upregulated by EX-527 in CCI mice.

**Conclusions:**

The discovery of the effect of SIRT1 on neuropathic pain in the VTA represents an important step forward in understanding the analgesic mechanisms of the VTA-NAc pathway.

## 1. Introduction

Chronic pain is a highly prevalent and intractable disease in the world, leading patients to wind up in a constant state of pain and contributing to high costs of health care and productivity loss [1–5]. Neuropathic pain, a kind of chronic pain, is defined by the International Association for the Study of Pain (IASP) as a pain arising after lesions or diseases, which affect the somatosensory system [6]. Epidemiological research studies show that the prevalence of neuropathic pain may be 7-8% in general population [7, 8], accounting for 20–25% of individuals with chronic pain [9]. Central sensitization, which augments responsiveness (amplification) of nociceptive neurons in the central nervous system to normal or subthreshold afferent inputs in the spinal cord and brain, may be a crucial mechanism and also a potential therapeutic target for neuropathic pain [10–13]. Mesolimbic reward circuitry, which is composed of dopaminergic (DA) neurons of the ventral tegmental area (VTA) and their projections to several brain regions, is pivotal for pain sensation [14–22]. Functional magnetic resonance images revealed that reward circuitry could be activated by noxious stimuli in healthy volunteers [23, 24]. The VTA, as a mesencephalic nucleus, is recently found to participate in pain sensation induced by mechanical stimulation, electrical foot shock, nerve injury, bone cancer, or formalin [15, 25–27]. The nucleus accumbens (NAc), a key projectional region of VTA DA neurons, plays an analgesic role in neuropathic pain [19, 20, 28–30].

Sirtuin 1 (SIRT1), a NAD^+^-dependent class III histone deacetylase, is deemed as a potential therapeutic target for several chronic diseases which are more common in elderly population, such as type 2 diabetes, cardiovascular diseases, cancers, age-related macular degeneration, and neurodegenerative diseases [31–35]. Additionally, SIRT1 has been reported to regulate anxiety, depressive and exploratory behaviors, and behaviors associated with drug abuse in the VTA and NAc [36–42]. Recent research studies illustrate that SIRT1 alleviates neuropathic pain induced by type 2 diabetes, Freund's adjuvant, bortezomib, paclitaxel, or chronic constriction injury (CCI) in the spinal cord and dorsal root ganglion [43–48]. The analgesic mechanisms of SIRT1 in the spinal cord and dorsal root ganglion are based on the epigenetic regulation of mGluR1/5, postsynaptic density protein 95 (PSD-95), growth-associated protein 43 (GAP43), c-fos, mammalian target of rapamycin (mTOR), nuclear factor-*κ*B (NF-*κ*B), interleukin-6 (IL-6), tumor necrosis factor-*α* (TNF-*α*), and inducible nitric oxide synthase (iNOS) expression [43–49]. Furthermore, immunohistochemistry staining showed that the expression of the *c-fos* gene, a neural marker of pain, was upregulated in the VTA-NAc pathway induced by CCI [22]. Intrathecal injection of c-fos antisense oligodeoxynucleotide to block c-fos gene expression decreased the nociceptive behaviors induced by formalin and Freund's adjuvant [50, 51].

Based on these findings, we reasoned that SIRT1 in the VTA-NAc pathway might mediate neuropathic pain through regulating Fos expression. Here, we showed that SIRT1 in the VTA-NAc pathway ameliorated neuropathic pain induced by sciatic nerve CCI.

## 2. Methods

### 2.1. Animals

**Figure 1:**
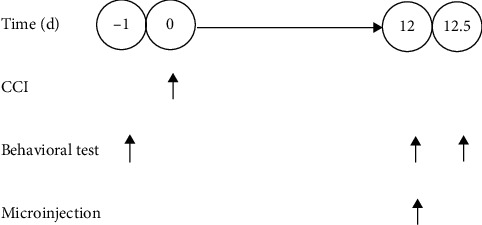
Experimental schedule. One day before the surgery, behavioral testing was performed to eliminate the mice exhibiting hyperalgesia or dysesthesia to pain. 12 days after the surgery, the CCI mice exhibiting hyperalgesia were microinjected with SRT1720, EX-527, or vehicle. 12 h after the microinjection, the effect of SRT1720 and EX-527 on neuropathic pain was indicated by behavioral testing.

The male mice, 18–22 g, were housed in a controlled condition (23 ± 2°C and 20%–30% humidity). The care and experimental procedures were performed in accordance with and approved by the Institutional Animal Care and Use Committee of Xuzhou Medical College. After 3 days of acclimatization, behavioral testing was performed to eliminate the mice exhibiting hyperalgesia or dysesthesia to thermal nociception, and the remaining mice were randomly assigned to different groups. In the following experiments (Figure 1 shows the experimental schedule), all efforts were made to minimize the number and suffering of mice.

### 2.2. Sciatic Nerve CCI Model

The CCI model was established according to the method of Bennett and Xie [52]. In brief, mice were anesthetized with pentobarbital sodium (35 mg/kg, i.p.). The left sciatic nerves were exposed at midthigh level and bluntly separated after skin cutting. Three ligatures were performed loosely on the sciatic nerves, proximally to the trifurcation using 5-0 silks at 1 mm interval. The sham surgery was performed with the sciatic nerve exposure on the left side without the hypodermis.

### 2.3. Behavioral Testing

Thermal nociception was assessed by measuring paw withdrawal latencies in response to thermal stimulation according to the protocol by Hargreaves et al. [53]. Mice were individually placed in chambers (7 × 9 × 11 cm) on an elevated glass platform and were allowed to acclimate to the environment for 1 h in a temperature-controlled (23 ± 2°C) and noise-free room. The high-intensity radiant lamp source placed underneath the glass plate (the thickness is about 3 mm) was focused onto the left plantar surface. The heat source was turned off when the paw withdrew. The interval time between the onset of radiant lamp application and the left paw withdrawal was recorded. The automatic cutoff time was 20 s to prevent tissue damages. The interval time of thermal stimulation was 5 min. The maximum and minimum of five values were deleted and the average of the remaining three values was deemed as the thermal withdrawal latency (TWL). 12 days after the surgery, the decrease in TWL in CCI mice indicated the success in the establishment of the neuropathic pain model.

### 2.4. Stereotaxic Surgery and Microinjection

Mice were anesthetized and positioned on the Stoelting Stereotaxic Instrument (David Kopf Instruments, Tujunga, CA). The drill coordinate for the Hamilton syringe needle was anteroposterior (AP) −3.5 mm, mediolateral (ML) ±0.5 mm, and dorsoventral (DV) −0.4 mm. 0.05 *µ*g/0.5 *µ*L SRT1720 (APExBIO, Houston, TX, USA) or 0.1 *µ*g/0.5 *µ*L EX-527 (APExBIO) was unilaterally infused at a rate of 0.1 *µ*L/min with a microinfusion pump (Harvard Apparatus, Holliston, MA, USA). Mice in the sham and CCI groups were injected with 0.5 *µ*L 1% dimethyl sulfoxide. An additional 5 min was allowed for diffusion and prevention of backflow.

### 2.5. Double Immunofluorescent Staining

The double immunofluorescent staining experiment was conducted on the brains of naive mice. Glial fibrillary acidic protein (GFAP), Iba-1, and NeuN are, respectively, regarded as the markers of the astrocyte, microglia, and neuron. After deeply anesthetized with pentobarbital sodium (40 mg/kg, i.p.), mice were perfused transcardially with 0.1 M precooling phosphate-buffered saline (PBS), followed by 4% paraformaldehyde (dissolved in PBS). The brains were removed and postfixed in 4% paraformaldehyde at 4°C for 24 h and then allowed to equilibrate in 30% sucrose (dissolved in PBS) until sinking to the bottom. The brains were sectioned to 30 *μ*m coronal sections by a cryostat (Leica, Wetzlar, Germany) at −20°C and stored in a frozen stock solution at −20°C. After being washed by PBS, the brain sections were blocked with the blocking buffer (3% goat serum and 1% Triton X-100) for 2 h. Thereafter, the sections were incubated with the anti-SIRT1 antibody (1 : 100; Abcam, Cambridge, UK) plus anti-GFAP antibody (1 : 100; Cell Signaling Technology Inc., Beverly, MA, USA), anti-Iba-1 antibody (1 : 100; Abcam), or anti-NeuN antibody (1 : 100; Cell Signaling Technology Inc.) respectively, for 48 h at 4°C. After being washed with PBS, the sections were incubated with the suitable secondary antibodies: Alexa Fluor® 594 Donkey anti-mouse IgG (Life Technologies, Carlsbad, CA, USA) or Alexa Fluor® 488 Donkey anti-Rabbit IgG (Life Technologies) in the darkness for 2 h. Thereafter, the sections were stained with 4′,6-diamidino-2-phenylindole (DAPI). Cellular colocalization was observed under a fluorescence microscope (Olympus, Tokyo, Japan).

### 2.6. Western Blotting Analysis

The tissues were homogenized in the lysis buffer containing a cocktail of protease inhibitors and phenylmethylsulfonyl fluoride (KeyGEN BioTECH, Jiangsu, China). After centrifugation at 12000 g for 15 minutes at 4°C, the supernatant was used for western blotting analysis. The protein concentration was measured with a BCA Protein Assay Kit (KeyGEN BioTECH). An equal amount of protein samples was separated by 10% sodium dodecyl sulfate-polyacrylamide gel electrophoresis (Lufei Bio, Jiangsu, China) and then transferred to the polyvinylidene fluoride membranes. The membranes were blocked with 5% nonfat milk. Thereafter, the membranes were incubated with the appropriate primary antibodies, including the anti-SIRT1 antibody (Abcam), anti-acetyl-histone 3 antibody (Cell Signaling Technology Inc.), anti-Fos antibody (Cell Signaling Technology Inc.), and anti-*β*-actin antibody (Abcam), at 4°C overnight, followed by incubation with the goat anti-rabbit or goat anti-mouse secondary antibody labeled with horseradish peroxidase (Jackson Immuno Research, West Grove, PA, USA). The expression of proteins was normalized to *β*-actin.

### 2.7. Coimmunoprecipitation Analysis

The VTA was lysed, and the total protein level was determined by the Bradford method. The lysates (with the identical concentration and amount of total proteins) were precleared by incubating the samples with Protein G agarose beads (Beyotime Biotechnology, Shanghai, China) on the shaker for 30 min at 4°C. After centrifugation, the supernatants were transferred to fresh 1.5 ml microfuge tubes. One microgram of the anti-SIRT1 antibody was added into each sample and incubated overnight at 4°C. Then, 100  *μ*L of 50% Protein G agarose beads was added into the tubes and incubated with the SIRT1-antibody complex at 4°C for 1 h. The beads were washed three times with the ice-cold lysis buffer and then incubated at 100°C for 5 min. Thereafter, the supernatant was subjected to sodium dodecyl sulfate-polyacrylamide gel electrophoresis. Western blot analysis was applied to analyze the combination between SIRT1 and its subunit acetyl-histone 3 by incubating the blots with these two primary antibodies at the same time.

### 2.8. Statistical Analysis

All data were presented as mean ± standard deviation (SD). Statistical analyses were conducted by one-way ANOVA followed by LSD (if the variance is equal) or the Dunnett T3 test (if the variance is not equal). Results were considered to be significantly different when *P* < 0.05.

## 3. Results

### 3.1. SIRT1 Localizes in the VTA Neuron but Not Astrocyte or Microglia

**Figure 2:**
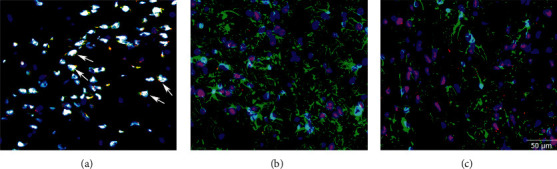
Localization of SIRT1 in the VTA of mice. Double immunofluorescent staining for SIRT1 (red) and NeuN (green), GFAP (green), or Iba-1 (green) was employed to identify the location of SIRT1 in the VTA. The nucleus was counterstained with DAPI (blue). Colocalization of SIRT1 and NeuN denotes that SIRT1 localizes in the nuclei of the neurons (white arrows). Scale bar = 50 *μ*m. (a) SIRT1/NeuN. (b) SIRT1/GFAP. (c) SIRT1/Iba-1.

The double immunofluorescent staining experiment was firstly conducted to investigate the location of SIRT1 in the VTA. GFAP, Iba-1, and NeuN are regarded as the markers of the astrocyte, microglia, and neuron, respectively. The results showed that SIRT1 (red) was almost colocalized with NeuN (green) in the VTA. However, overlaps of SIRT1 (red) with GFAP (green) or Iba-1 (green) were rarely seen (Figure 2). These results indicate that SIRT1 may be involved in regulating neuronal function in the VTA.

### 3.2. Upregulation of SIRT1 Attenuates Pain Behavior and VTA Neuronal Activation in CCI Mice

**Figure 3:**
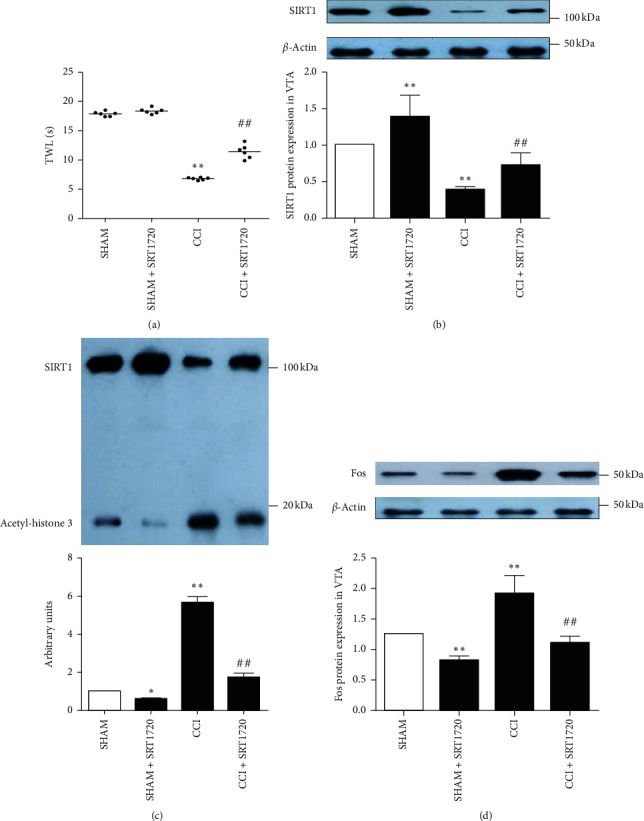
Effects of SRT1720 on pain behavior and VTA neuronal activation in CCI mice. CCI mice exhibited thermal hyperalgesia in the affected hind paws, which was relieved by SRT1720 (a). The expression of SIRT1 in the contralateral VTA was inhibited by CCI but upregulated by SRT1720 (b). The increased level of acetyl-histone 3 in the contralateral VTA induced by CCI was downregulated by SRT1720 (c). The increased expression of Fos in the contralateral VTA neurons induced by CCI was inhibited by SRT1720 (d). Data are presented as mean ± SD. *n* = 6 mice per group (*n* = 3 in the coimmunoprecipitation experiment). ^*∗*^*P* < 0.05, ^*∗∗*^*P* < 0.01 versus the sham-operated group, ^#^*P* < 0.05, and ^##^*P* < 0.01 versus the CCI group.

To investigate the role of SIRT1 in neuropathic pain, SRT1720, a selective SIRT1 agonist, was microinjected into the contralateral VTA. 12 h later, a behavioral test was performed. CCI mice exhibited thermal hyperalgesia in the affected hind paws, which was mitigated by SRT1720 (Figure 3(a)). Contrary to the changes of pain behavior, the downregulated expression of SIRT1 in the contralateral VTA of CCI mice was upregulated by SRT1720 (Figure 3(b)). Furthermore, the modifications of histone 3 are involved in the epigenetic modulations of gene expression. The acetylation of histone 3, regulated by the histone deacetylases and histone acetyltransferases, is involved in the initiation and maintenance of pain [44, 54]. Coimmunoprecipitation analysis revealed that the level of acetyl-histone 3, a substrate of SIRT1, was elevated in the contralateral VTA of CCI mice, and the degree of elevation was decreased by SRT1720 (Figure 3(c)). It is known that the Fos protein, the product of the c-fos immediate early gene, has been deemed as a marker for neuronal activation in the central nervous system [55–57]. Western blotting analysis revealed that SRT1720 alleviated the increased expression of Fos in the contralateral VTA of CCI mice (Figure 3(d)). These results indicate that upregulating the expression of SIRT1 by SRT1720 may ameliorate hyperalgesia and inhibit the VTA neuronal activation induced by CCI.

### 3.3. Downregulation of SIRT1 Aggravates Pain Behavior and VTA Neuronal Activation in CCI Mice

**Figure 4:**
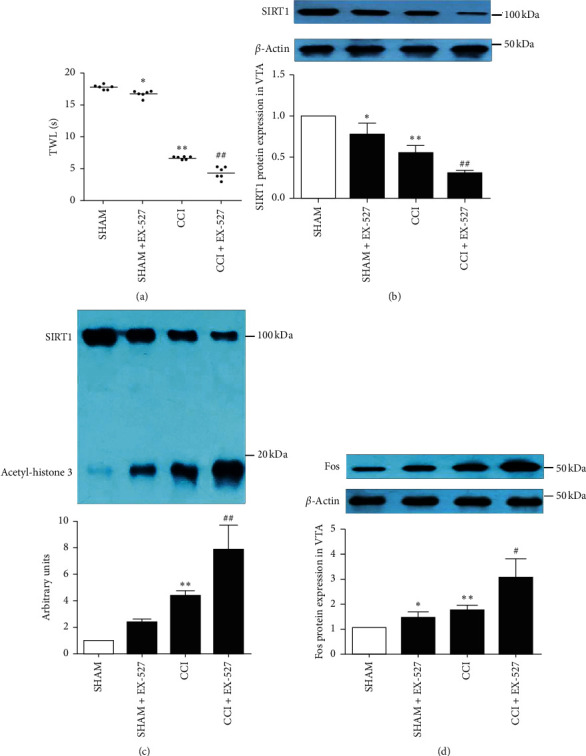
Effects of EX-527 on pain behavior and VTA neuronal activation in CCI mice. EX-527 significantly reduced the TWL in sham-operated mice and further reduced the TWL in CCI mice (a). The expression of SIRT1 in the contralateral VTA of sham-operated and CCI mice was inhibited by EX-527 (b). The level of acetyl-histone 3 in the contralateral VTA of sham-operated and CCI mice was increased by EX-527 (c). The expression of Fos in the contralateral VTA of sham-operated and CCI mice was upregulated by EX-527 (d). Data are presented as mean ± SD. *n* = 6 mice per group (*n* = 3 in the coimmunoprecipitation experiment). ^*∗*^*P* < 0.05, ^*∗∗*^*P* < 0.01 versus the sham-operated group, ^#^*P* < 0.05, and ^##^*P* < 0.01 versus the CCI group.

To validate the effect of SIRT1 on neuropathic pain in the VTA, EX-527, a selective SIRT1 inhibitor, was employed in this study. EX-527 significantly decreased the TWL in sham-operated mice and further decreased the TWL in CCI mice (Figure 4(a)). EX-527 significantly inhibited the expression of SIRT1 in the contralateral VTA in sham-operated and CCI mice (Figure 4(b)). Moreover, the coimmunoprecipitation experiment indicated that EX-527 significantly increased the level of acetyl-histone 3 in the contralateral VTA of sham-operated and CCI mice (Figure 4(c)). In addition, western blotting analysis revealed that EX-527 significantly upregulated the expression of Fos in the contralateral VTA of sham-operated and CCI mice (Figure 4(d)). These results indicate that downregulating the expression of SIRT1 by EX-527 may exacerbate hyperalgesia and activate the VTA neurons in sham-operated and CCI mice.

### 3.4. SIRT1 in the VTA Inhibits the NAc Neuronal Activation in CCI Mice

**Figure 5:**
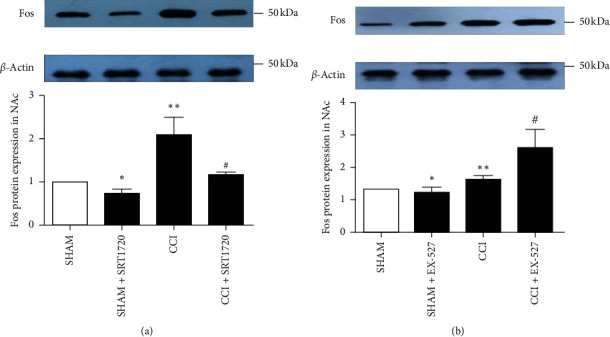
Effect of SIRT1 on the activation of NAc neurons in CCI mice. The increased expression of Fos in the contralateral NAc neurons induced by CCI was inhibited by SRT1720 (a). EX-527 microinjected in the VTA significantly induced the expression of Fos in the contralateral NAc of sham-operated and CCI mice (b). Data are presented as mean ± SD. *n* = 6 mice per group. ^*∗*^*P* < 0.05, ^*∗∗*^*P* < 0.01 versus the sham-operated group, ^#^*P* < 0.05, and ^##^*P* < 0.01 versus the CCI group.

The NAc is a key projectional region of VTA neurons, playing an analgesic role in neuropathic pain. To further probe the analgesic mechanism of SIRT1 in the VTA, western blotting analysis was conducted. SRT1720 microinjected in the VTA alleviated the increased level of Fos in the contralateral NAc neurons induced by CCI (Figure 5(a)); nevertheless, EX-527 significantly aggravated the expression of Fos (Figure 5(b)). These results demonstrate that SIRT1 in the VTA mediates neuropathic pain, maybe through inhibiting the activation of neurons in the NAc, which is the projectional region of VTA DA neurons.

## 4. Discussion

Central sensitization has been identified as a crucial mechanism for regulating hyperalgesia and also a potential therapeutic target for neuropathic pain, through augmenting responsiveness of nociceptive neurons in the central nervous system to normal or subthreshold afferent inputs in the spinal cord and the brain. Mounting evidence reveals that mesolimbic reward circuitry, which is composed of DA neurons of VTA and their projections to NAc, is pivotal for pain sensation [14–22]. As an NAD^+^-dependent histone deacetylase, SIRT1 is extensively studied in several senile diseases including type 2 diabetes, cardiovascular diseases, cancers, age-related macular degeneration, and neurodegenerative diseases. Moreover, SIRT1 has been implicated in regulating anxiety, depressive and exploratory behaviors, and the behaviors associated with drug abuse in the VTA and NAc [36–42]. Recently, research studies illustrate the analgesic effect of SIRT1 on neuropathic pain induced by Freund's adjuvant, bortezomib, chronic constriction injury, and paclitaxel in the spinal cord and the dorsal root ganglion [43, 45–48]. Our previous research has shown that SIRT1-mediated epigenetic regulation of mGluR1/5 expressions were involved in the development of neuropathic pain in type 2 diabetic rats [44]. The objective of the present study was to explore the possible role of SIRT1 in neuropathic pain induced by CCI in the VTA-NAc pathway.

In this study, we firstly conducted a double immunofluorescent staining experiment to investigate the location of SIRT1 in the VTA. The results showed that SIRT1 almost colocalized with NeuN, indicating that SIRT1 might regulate neuronal functions in the VTA. In the following experiments, we found that the expression of SIRT1 was inhibited in the contralateral VTA of CCI animals. Moreover, established thermal hyperalgesia was attenuated by SRT1720 but further exacerbated by EX-527, indicating that SIRT1 in the VTA may participate in analgesia. Recent research studies have substantiated that acetyl-histone 3 might be a key analgesic target for SIRT1 in neuropathic pain induced by CCI and type 2 diabetes [44, 54]. Our data suggested that acetyl-histone 3 in the VTA was involved in the analgesic effect of SIRT1 in CCI models. The Fos protein has been considered as the marker and causal contributor for neuronal activation in the central nervous system [50, 51, 54–56]. We found that the elevated level of the Fos protein in both VTA and NAc was decreased by SRT1720 but further increased by EX-527 in CCI mice, indicating that SIRT1 may inhibit the activation of neurons in the VTA and then inhibit the activation of neurons in the NAc, which is a key projectional region of VTA DA neurons.

## 5. Conclusions

Taken together, the present study manifests that the decrease in SIRT1 in the VTA may contribute to the neuropathic pain and the activation of neurons in the VTA and NAc in CCI mice. Upregulation of SIRT1 in the VTA can alleviate neuropathic pain and inhibit the activation of neurons in the VTA and NAc induced by CCI. Our findings may provide a novel insight for the analgesic effect of the VTA-NAc pathway and provide useful information for developing new strategies to treat neuropathic pain.

## Data Availability

The data used to support the findings of this study are available from the corresponding author upon request.
